# Outbreak investigation of lead neurotoxicity in children from artificial jewelry cottage industry

**DOI:** 10.1186/s12199-019-0777-9

**Published:** 2019-05-10

**Authors:** Akhil D. Goel, Rohini V. Chowgule

**Affiliations:** 1 Department of Community Medicine and Family Medicine, All India Institute of Medical Sciences, Jodhpur, India; 2 Indian Institute of Environmental Medicine, Kasturba Hospital Mumbai, Building no. 12, Mumbai, India

**Keywords:** Heavy metal poisoning, Case control study, Inhalation exposure

## Abstract

**Background:**

Although lead neurotoxicity is a known phenomenon, it can often be missed at a primary or secondary care level especially if detailed environmental exposure history is missed.

**Methods:**

This is an outbreak investigation where we observed 15 pediatric cases with neurologic signs and symptoms clustered in a slum area known for an unorganized artificial jewelry industry. Their clinical, biochemical, and epidemiological features were compared with 14 other children from the same region reporting with non-neurological symptoms who were considered as unmatched controls.

**Results:**

Cases with neurological manifestations had a higher in-house lead smelting activity [OR 7.2 (95% CI 1.4–38.3)] as compared to controls. Toddlers below 3 years of age were more vulnerable to the effects of lead.

**Conclusion:**

This study emphasizes that many focal sources of lead poisoning still remain especially in the unorganized sector. In cases presenting with unexplained neurotoxicity, specific occupational and environmental inquiry for chemical poisoning, with special consideration for lead, should be actively pursued.

**Electronic supplementary material:**

The online version of this article (10.1186/s12199-019-0777-9) contains supplementary material, which is available to authorized users.

## Key messages

- Inhaled aerosolized lead can lead to acute neurotoxicity.
- Workers and their family members engaged in the unorganized sector often are exposed to occupational and/ or environmentally harmful chemicals and their early diagnosis can be challenging.

## Introduction

Lead toxicity is turning out to be a pernicious health hazard of all age groups with major socio-economic implications. WHO estimates that there are 120 million people worldwide who are lead poisoned (i.e., have a blood lead level (BLL) greater than 10 μg/dL) [1]. Although leaded gasoline, paints, toys, drinking water, and other major sources are being faded out, there still exist pockets of lead exposure which need deliberation and study.

While developed countries have improvised greatly on these conventional sources of lead exposure and are moving towards establishing the effects of lower BLL [2], India is still struggling with building the awareness of lead as a potentially cumulative neuro-psycho-poison. Unregulated industrial emissions, absence of strict labor, and environmental safety laws with unemployment and child labor are providing a good means for encouraging a lead epidemic.

We observed a number of cases reporting with convulsions and drowsiness from one particular area which had an unorganized artificial jewelry cottage industry and investigated this further through the current study.

## Materials and methods

Mumbai, like many other urban metropolitan cities, houses multiple slum pockets where people are engaged in various small scale and often unregulated occupations. During a routine clinical audit in 2011 of a primary child care clinic in a northwestern suburb of Mumbai, we observed that a number of children residing in a small slum pocket reported with neurological symptoms of varying severity. The residents here stay in shanties measuring about 3.7 × 3.7 m which are ill-ventilated enclosures with an average four to five persons living in them and also housing a kerosene stove at one corner which was used for smelting lead to make artificial jewelry. To epidemiologically investigate these clustering of cases, a case-control study was conducted. Cases were children with neurological complaints like convulsions (focal or generalized) and drowsiness (lethargic or increased sleepiness) as reported by parents were continuously recruited from a primary child care clinic over a 6-month period. Controls were those without neurological complaints recruited actively by visiting the neighborhood of cases (and not from the houses of cases). Both cases and controls were recruited simultaneously during the post-monsoon period of October to December, 2011. The recruiters for controls can be considered to be blinded as they would routinely visit the community and refer children to a child care clinic for health services like immunization and other morbidities. As it was a small slum pocket, we could not do randomization, age/gender matching or recruit a higher number of controls. The cases and controls can be considered comparable for socioeconomic status and other environmental exposures from the same vicinity.

The parents of the study subjects were interviewed for a lead exposure history of lead smelting inside the house, house painting in a past year, use of *surma* (traditional eye cosmetic involving lead), and pica for the analysis. In addition, the following information was collected for exclusion criteria: ayurvedic/homeopathic drug history, family history, socioeconomic history, and occupational history. A written informed valid consent was taken from the parents of these children along with assent of those children ≥ 7 years of age. Institutional ethical committee clearance was obtained from the Indian Institute of Environmental Medicine, Mumbai, for the study. Detailed hematological assays (hemoglobin and complete blood counts), cerebrospinal fluid analysis (CSF) (proteins, sugar, and cell cytology), and magnetic resonance imaging (MRI) were used to investigate into the causes of clinical presentations of convulsions and drowsiness. One child was excluded because of the diagnosis of tuberculous meningitis confirmed on CSF analysis. Those with an identifiable infectious, metabolic, or systemic cause were excluded from the study. All the children were examined for their age, sex, weight, hemoglobin (Hb) levels, and blood lead levels (BLL).

For BLL measurement, 1 mL of blood sample was collected in heparinized polypropylene tubes and wet digested using electronic graded nitric acid and perchloric acid. Digested samples were carefully made up to 10 mL using 0.25% nitric acid. After completion of wet ashing, the lead content in the sample was measured using differential pulse anodic stripping voltammetry which is highly sensitive in detecting as low as 10^−7^ to 10^−9^ concentrations of lead. EG&G Princeton applied research model 394 analyzer was used. A static mercury drop electrode assembly (Parc-303 A) was used with a parc-305 stirrer. Voltammograms were recorded with a par x-y recorder (model RE0074). This method can measure blood lead levels in nanograms and is thus more sensitive than atom absorptiometry. This test was performed by trained laboratory technicians at the environmental lab located at Indian Institute of Environmental Medicine, Mumbai.

Statistical Package for Social Sciences (SPSS) v.19 has been used for statistical analysis. Nominal variables like the presence of neurological symptoms and the presence of lead smelting inside the house were described using frequencies and percentages and compared using Fisher’s exact test. Quantitative variables like blood lead levels were described using mean and standard deviation and compared using the Mann-Whitney *U* test. A *p* value of < 0.05 was considered significant. The dataset supporting the conclusion of this article is available in Additional file 1.

## Results

We studied 29 children (15 cases, 14 controls) aged between 0.5 and 13 years belonging to the slum pockets of northwestern suburbs of Mumbai with a mushrooming unorganized artificial jewelry cottage industry. The residents here stay in shanties measuring about 3.7 × 3.7 m which are ill-ventilated enclosures with an average four to five persons living in them and also housing a kerosene stove at one corner which was used for smelting lead to make artificial jewelry.

The cases with neurological symptoms included seizures (*n* = 12) and drowsiness (*n* = 3). The overall mean ages of cases (3.9 ± 4.1 years) and of controls (5.3 ± 1.9 years) were not significantly different (*p* = 0.27). Gender distribution was also similar with 60% cases and 50% controls being males (*p* = 0.59) (Table 1). None of these 29 children had a history of any ayurvedic/homeopathic drugs, infections, significant past medical illness, or relevant family history of neurological disease.

**Table 1:** Characteristics of cases and controls

	Cases (*n* = 15)	Controls (*n* = 14)	Odds ratio	*p* value
Demography				
Age, mean ± S.D. (years)	3.9 ± 4.1	5.3 ± 1.9	–	0.56^a^
Male, freq. (%)	9 (60.0%)	7 (50.0)	–	0.59^b^
Exposure history				
Lead smelting inside house	12 (80.0%)	5 (35.7%)	7.20 [1.40–38.30]	*0.04* ^b^
House painting/renovation in past year	1 (6.7%)	2 (14.3%)	0.43 [0.03–5.30]	0.59^b^
Use of *surma*^c^	1 (6.7%)	3 (21.4%)	0.26 [0.02–2.88]	0.33^b^
Pica, freq	3 (20.0%)	2 (14.3%)	1.50 [0.21–10.65]	1.00^b^
Investigations				
Weight, mean ± S.D. (kg)	10.2 ± 5.7	14.4 ± 4.6	**–**	*0.03* ^a^
Hb, mean ± S.D. (gm/dL)	8.5 ± 1.4	10.8 ± 2.1	**–**	*0.003* ^a^
BLL, mean ± S.D. (μg/dL)	42.6 ± 22.5	8.7 ± 1.2	**–**	*< 0.001* ^a^

All figures in round parentheses represent column-wise percentages. Figures in square parentheses represent 95% CI

*Abbreviations*: *Hb* hemoglobin, *BLL* blood lead levels

^a^*p* values calculated using the Mann-Whitney *U* test

^b^*p* values calculated using the Fisher exact test

^c^*Surma* is the local word for kohl used as a traditional eye cosmetic and has been known to have high lead

The mean BLL of cases was 42.6 ± 22.5 μg/dL (range 16.6–85.4) which was significantly higher in exposed children than in controls 8.7 ± 1.2 μg/dL (range 7.0–10.2) (*p* < 0.001). All the cases were treated symptomatically and referred to a tertiary care center for chelation therapy. Twelve out of 15 (80.0%) cases gave a history of lead smelting activities inside their homes as compared to only 5 out of 14 (35.7%) controls [OR 7.2 (95% CI 1.4–38.3)]. Other potential risk factors for lead exposure like painting of house, pica, or use of *surma* were low in both cases and controls.

**Fig. 1:**
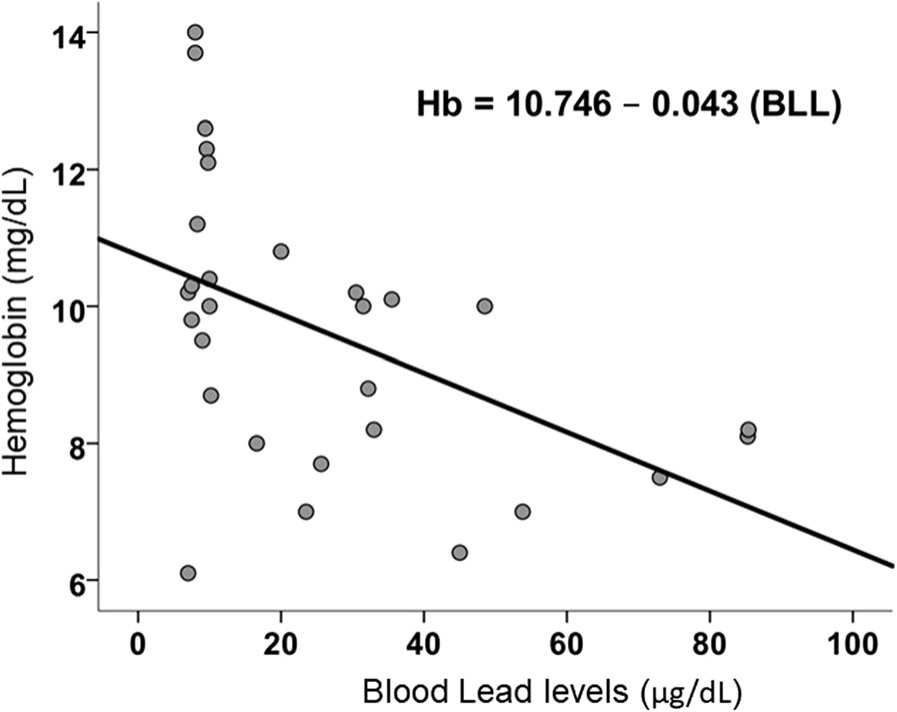
Correlation plot showing the relationship between blood lead levels and hemoglobin

As compared to controls, cases had a significantly higher BLL (*p* < 0.001), a significantly lowered Hb (*p* = 0.002), and a significantly lowered weight (*p* = 0.038) (Table 1). There was a significant negative correlation between BLL and Hb which showed that the BLL linearly increased with decreases on the Hb (Pearson’s *r* = − 0.537, *p* = 0.003) (Fig. 1).

The 12 out of 15 cases who had lead smelting units inside their houses could be considered to be exposed to aerosolized lead. Similarly, 5 out of 14 controls with lead smelting undertaken within their houses had a significantly higher BLL as compared to those who did not (*p* = 0.003). In addition, the Hb and weight of children with lead smelting inside their houses were statistically lower than that of those who did not, respectively (Hb: *P* < 0.003 and weight: *P* < 0.03).

Among the cases, BLL in toddlers below 3 years (46.9 ± 27.2 μg/dL) was statistically similar to that of cases above 3 years (36.2 ± 12.6 μg/dL) [*p* = 0.39]. However, the Hb of toddlers below 3 years (7.98 ± 1.06 g%) was significantly lower from cases above 3 years (9.37 ± 1.45 g%) [*p* = 0.05]. On linear regression, BLL (*β* = − 0.361, *p* = 0.049, *R*^2^ = 0.330) continued to significantly influence Hb after adjusting for age. This may reflect the higher vulnerability of younger children suffering from anemia caused by lead exposure (Table 2).

**Table 2:** Children below 3 years of age show significantly greater anemia for similar BLL among cases (*n* = 15)

	Control			Case			Total		
	> 3 years (*n* = 11)	≤ 3 years (*n* = 3)	*p* value	> 3 years (*n* = 6)	≤ 3 years (*n* = 9)	*p* value	> 3 years (*n* = 17)	≤ 3 years (*n* = 12)	*p* value
BLL, mean ± S.D. (μg/dL)	9.0 ± 1.0	7.3 ± 0.6	0.02	36.2 ± 12.6	46.9 ± 27.2	0.39	18.6 ± 15.1	37.0 ± 29.3	0.04
Hb, mean ± S.D. (gm/dL)	11.0 ± 1.6	10.0 ± 3.8	0.49	9.4 ± 1.5	8.0 ± 1.1	*0.054*	10.4 ± 1.7	8.5 ± 2.1	0.01

*Abbreviations*: *Hb* hemoglobin, *BLL* blood lead levels

## Discussion

This study epidemiologically links the otherwise known association of lead causing neurotoxicity in children. But the mean BLL of 42.6 ± 22.5 μg/dL seen in our cases with neurological symptoms was much less than the level of 80 μg/dL which is suggested cutoff when neurotoxicity is likely to occur. This may indicate acute lead toxicity most probably from ambient aerosolized lead and ill-ventilated smelting areas. Lead smelting within the house was the most important risk factor associated with the cases. Also, lead appeared to affect the younger children more than the elder ones. One reason for this could be prolonged exposure in the fetus right from the antenatal period; however, there is conflicting evidence both supporting [3] and refuting [4] the importance of antenatal lead exposure.

The artificial jewelry cottage industry is one of the many examples which make us realize the gradually teeming up problem of lead toxicity in unsuspecting locations. And by its virtue of being unorganized and small scale, it is not yet on the radar of government for further prevention of this occupation in its current form. The aerosolized lead poses a risk to not only indoor residents of the shanties but also the overcrowded community at large. With phasing out of the leaded fuel and public health regulations, we have now been able to reduce the external environmental lead exposure. Many research studies in different parts of India have reported a declining lead concentration in various environmental components especially in the current unleaded petrol phase [5]. But the problem of lead toxicity appears to be more complicated.

A large body of literature is available on the effects of lead and lead encephalopathy [6–9]. More than 70% of the total burden of lead in children is concentrated in neurological tissue. This is accompanied by leakiness of the blood-brain barrier [10, 11], thus making children more prone to lead toxicity. Our study corroborates the well-known fact that lead encephalopathy occurs at BLL > 70 μg/dL [12]. But we also see convulsions and drowsiness even at BLL well below 80 μg/dL. This is probably due to the lower body surface area of children and perhaps the lower socioeconomic status with accompanying malnutrition which predisposes them to encephalopathy at much lower lead levels. Also, the weight of all the children in this study was below the ideal weight for their age probably due to varied factors like poor nutrition with lead exposure being a potential synergistic contributor. On multiple logistic regression, after adjusting for age, weight was found to be significantly lower in cases than in controls [OR = 0.69; 95% CI 0.48–0.99].

The mean BLL of cases in our study is three times higher than the other Indian studies [13–15]. This can again be attributed to the obvious parental occupational history of making artificial jewelry by smelting lead inside the residence itself. There were no other significant discernible causes seen in our study population.

Lead-induced anemia is produced principally by two mechanisms: impairment in heme biosynthesis and increased rate of red blood cell destruction [8, 16]. Accompanying malnutrition and iron deficiency anemia are also known to enhance the susceptibility to lead [17, 18]. All the 29 children had low Hb mostly due to the low socioeconomic status with poor nutrition. Poor socioeconomic status is a known risk factor for anemia and undernutrition. However, Hb was significantly lowered (*p* < 0.05) in children < 3 years as compared to those > 3 years (Table 2). This reflects a higher vulnerability in very young children at similar lead levels. Similar findings were also reported by Jain et.al. who analyzed the National Family Health Survey 1998–1999 data and found that children with higher lead levels had a significantly higher association with anemia in the below 3-year age group [19]. We thus need to look into redefining lead levels, especially in the young pediatric population.

This study cannot establish a direct cause-effect relationship between the artificial jewelry occupation and the neurological problems or anemia in the children. The small sample size of this epidemiological investigation might limit the statistical quality of the analysis. The current study also did not measure indoor air samples for lead which could help in strengthening the cause-effect relationship. But the evidence is strong enough to propose preventive measures like the isolation of children from the workplace, use of barrier protection while working, and adequate hand and environmental hygiene. These issues were discussed with the artificial jewelry workers in a group and door-to-door awareness campaign. They said they knew that their job is killing their dear ones but had no other option to make a living! Nonetheless, it is prudent to exercise caution in both manufacturing and consuming these low-cost jewelry items.

## Conclusion

This study highlights the health risks due to inhalation of aerosolized lead in the artificial jewelry cottage industries especially among the socio-economically deprived unsuspecting children in the shanties of Mumbai and the need for preventive hygiene intervention to reduce the toxic exposure. It re-emphasizes that in pediatric cases with unexplained neurological symptoms, the diagnosis of environmental lead poisoning should always be considered. Further, younger children are far more susceptible than their older counterparts to similar BLL indicating higher vulnerability in them. Thus, even for apparently simple cases of anemia- and malnutrition-specific inquiry into the occupational and environmental history should be actively sought after.

From the wider public health and policy-making perspective, phasing out of lead from gasoline has been a critical first step in decreasing worldwide blood lead concentrations. However, especially in a large and diverse country like India, many focal sources of lead poisoning still remain, such as lead from mining and smelting, flour mills, lead-glazed ceramics, and battery repair and recycling. The challenge now is to implement effective national and regional efforts to identify the specific sources of lead and formulate preventive and interventional measures to tackle the associated morbidity.

## Additional file

Additional file 1: Dataset (DOCX 14 kb)

## Abbreviations

BLL: Blood lead level
CSF: Cerebrospinal fluid analysis
Hb: Hemoglobin
MRI: Magnetic resonance imaging

## References

[1] Fewtrell L, Kaufmann R, Prüss-Üstün A. Lead: assessing the environmental burden of disease at national and local levels. World Health Organization - Protection of the Human Environment, Geneva 2003, WHO Library [Internet]. Prüss-Üstün A, Campbell-Lendrum D, Corvalán C, Woodward A, editors. WHO Environmental Burden of Disease Series, No. 2; 2003 [cited 2017 Nov 17]. Available from: http://www.who.int/quantifying_ehimpacts/publications/en/leadebd2.pdf?ua=1.

[2] Centers for Disease Control and Prevention (CDC). Blood lead levels--United States, 1988–1991. MMWR Morb Mortal Wkly Rep [Internet]. 1994 May 27 [cited 2017 Nov 17];43(30):545–8. Available from: http://www.ncbi.nlm.nih.gov/pubmed/15917736 8035771

[3] Zhu M, Fitzgerald EF, Gelberg KH, Lin S, Druschel CM (2010). Maternal low-level lead exposure and fetal growth. Environ Health Perspect.

[4] Tatsuta N, Kurokawa N, Nakai K, Suzuki K, Iwai-Shimada M, Murata K (2017). Effects of intrauterine exposures to polychlorinated biphenyls, methylmercury, and lead on birth weight in Japanese male and female newborns. Environ Health Prev Med.

[5] Singh AK, Singh M (2006). Lead decline in the Indian environment resulting from the petrol-lead phase-out programme. Sci Total Environ.

[6] Canfield RL, Henderson CR, Cory-Slechta DA, Cox C, Jusko TA, Lanphear BP (2004). Intellectual impairment in children with blood lead concentrations below 10 μg per deciliter. J Dev Behav Pediatr.

[7] Goldstein GW (1992). Neurologic concepts of lead poisoning in children. Pediatr Ann.

[8] Goyer RA, Rhyne BC (1973). Pathological effects of lead. Int Rev Exp Pathol.

[9] Oberle MW (1969). Lead poisoning: a preventable childhood disease of the slums. Science..

[10] Skerfving S, Nilsson U, Schütz A, Gerhardsson L (1993). Biological monitoring of inorganic lead. Scand J Work Environ Health.

[11] Shi LZ, Zheng W (2007). Early lead exposure increases the leakage of the blood-cerebrospinal fluid barrier, in vitro. Hum Exp Toxicol.

[12] Abadin H, Ashizawa A, Stevens Y-W, Llados F, Diamond G, Sage G, et al. Toxicological profile for Lead. US public heal Serv agency toxic Subst dis Regist [internet]. 2007 [cited 2017 Nov 17];(august):582. Available from: https://www.atsdr.cdc.gov/ToxProfiles/tp.asp?id=96&tid=22. 24049859

[13] Kumar A, Dey PK, Singla PN, Ambasht RS, Upadhyay SK (1998). Blood lead levels in children with neurological disorders. J Trop Pediatr.

[14] Ahamed M, Fareed M, Kumar A, Siddiqui WA, Siddiqui MKJ (2008). Oxidative stress and neurological disorders in relation to blood lead levels in children. Redox Rep.

[15] Patel A, Athawale A (2009). Blood lead levels in children with encephalopathy. Indian Pediatr.

[16] Schwartz J, Landrigan PJ, Baker EL, Orenstein WA, Von Lindern IH (1990). Lead-induced anemia: dose-response relationships and evidence for a threshold. Am J Public Health.

[17] Kwong W, Friello P, Semba R (2004). Interactions between iron deficiency and lead poisoning: epidemiology and pathogenesis. Sci Total Environ.

[18] Kim H-S, Lee S-S, Hwangbo Y, Ahn K-D, Lee B-K (2003). Cross-sectional study of blood lead effects on iron status in Korean lead workers. Nutrition..

[19] Jain NB, Laden F, Guller U, Shankar A, Kazani S, Garshick E (2005). Relation between blood Lead levels and childhood Anemia in India. Am J Epidemiol.

