# Supplementary material for: Outbreak investigation of lead neurotoxicity in children from artificial jewelry cottage industry
Source: Environ Health Prev Med. 2019 May 10;24:30. doi: 10.1186/s12199-019-0777-9 (PMC6511187; doi:10.1186/s12199-019-0777-9)
Supplement: Supplementary file 1 — Dataset (DOCX 14 kb) [file 12199_2019_777_MOESM1_ESM.docx]

Additional File 1: Dataset

| **S. No.** | **Age** | **Group** | **Sex** | **Weight (kg)** | **Hb (g/dl)** | **BLL (µg/dl)** | **Lead Smelting in house** |
| --- | --- | --- | --- | --- | --- | --- | --- |
| 1 | 1.0 | Case | Female | 4.0 | 6.4 | 45.0 | Yes |
| 2 | 1.0 | Case | Male | 6.1 | 8.1 | 85.3 | Yes |
| 3 | 2.5 | Case | Female | 10.3 | 7.7 | 25.6 | Yes |
| 4 | 1.0 | Case | Male | 5.0 | 8.2 | 85.4 | Yes |
| 5 | 13.0 | Case | Male | 23.0 | 10.0 | 31.5 | Yes |
| 6 | 12.0 | Case | Female | 20.0 | 7.0 | 53.8 | Yes |
| 7 | 5.0 | Case | Male | 15.0 | 10.0 | 48.5 | Yes |
| 8 | 8.0 | Case | Female | 12.0 | 10.8 | 20.0 | Yes |
| 9 | 3.0 | Case | Male | 7.5 | 10.1 | 35.5 | Yes |
| 10 | 5.0 | Case | Male | 14.6 | 10.2 | 30.5 | No |
| 11 | 1.0 | Case | Female | 6.9 | 7.0 | 23.5 | Yes |
| 12 | 4.0 | Case | Female | 8.6 | 8.2 | 33.0 | Yes |
| 13 | 0.6 | Case | Male | 7.1 | 8.0 | 16.6 | No |
| 14 | 0.5 | Case | Male | 5.8 | 8.8 | 32.2 | No |
| 15 | 2.0 | Case | Male | 7.0 | 7.5 | 73.0 | Yes |
| 16 | 3.5 | Control | Female | 16.0 | 8.7 | 10.2 | Yes |
| 17 | 8.0 | Control | Female | 16.9 | 10.0 | 10.0 | No |
| 18 | 6.0 | Control | Male | 15.3 | 9.5 | 9.0 | No |
| 19 | 3.0 | Control | Male | 6.5 | 6.1 | 7.0 | Yes |
| 20 | 5.0 | Control | Female | 18.3 | 9.8 | 7.5 | Yes |
| 21 | 7.0 | Control | Female | 19.7 | 14.0 | 8.0 | Yes |
| 22 | 2.0 | Control | Female | 9.9 | 10.2 | 7.0 | No |
| 23 | 3.0 | Control | Male | 4.0 | 13.7 | 8.0 | No |
| 24 | 5.0 | Control | Male | 14.7 | 10.4 | 10.0 | No |
| 25 | 6.0 | Control | Male | 15.8 | 12.6 | 9.4 | No |
| 26 | 6.0 | Control | Female | 16.0 | 12.3 | 9.6 | No |
| 27 | 6.0 | Control | Male | 16.0 | 12.1 | 9.8 | No |
| 28 | 9.0 | Control | Female | 19.5 | 10.3 | 7.5 | Yes |
| 29 | 5.0 | Control | Male | 13.2 | 11.2 | 8.3 | No |
